# Cardiac Pathology and Molecular Epidemiology by Avian Leukosis Viruses in Japan

**DOI:** 10.1371/journal.pone.0086546

**Published:** 2014-01-23

**Authors:** Sayuri Nakamura, Kenji Ochiai, Akihiro Ochi, Hiroki Yabushita, Asumi Abe, Sayaka Kishi, Yuji Sunden, Takashi Umemura

**Affiliations:** 1 Laboratory of Comparative Pathology, Graduate School of Veterinary Medicine, Hokkaido University, Sapporo, Japan; 2 Miho Training Center, Japan Racing Association, Utsunomiya, Japan; 3 Medical School, Okayama University, Okayama, Japan; 4 Kenpoku Meat Hygiene Inspection Institute, Tochigi Prefecture, Ohtawara, Japan; The Scripps Research Institute, United States of America

## Abstract

Epidemiological studies suggest that retroviruses, including human immunodeficiency virus type 1, are associated with cardiomyopathy and myocarditis, but a causal relationship remains to be established. We encountered unusual cardiomyocyte hypertrophy and mitosis in Japanese native fowls infected with subgroup A of the avian leukosis viruses (ALVs-A), which belong to the genus *Alpharetrovirus* of the family *Retroviridae* and mainly induce lymphoid neoplasm in chickens. The affected hearts were evaluated by histopathology and immunohistochemistry, viral isolation, viral genome sequencing and experimental infection. There was non-suppurative myocarditis in eighteen fowls and seven of them had abnormal cardiomyocytes, which were distributed predominantly in the left ventricular wall and showed hypertrophic cytoplasm and atypical large nuclei. Nuclear chains and mitosis were frequently noted in these cardiomyocytes and immunohistochemistry for proliferating cell nuclear antigen supported the enhancement of mitotic activity. ALVs were isolated from all affected cases and phylogenic analysis of *env*SU genes showed that the isolates were mainly classified into two different clusters, suggesting viral genome diversity. *In ovo* experimental infection with two of the isolates was demonstrated to cause myocarditis and cardiomyocyte hypertrophy similar to those in the naturally occurring lesions and cardiac hamartoma (rhabdomyoma) in a shorter period of time (at 70 days of age) than expected. These results indicate that ALVs cause myocarditis as well as cardiomyocyte abnormality in chickens, implying a pathogenetic mechanism different from insertional mutagenesis and the existence of retrovirus-induced heart disorder.

## Introduction

Retroviruses cause a variety of illnesses such as leukemia, acquired immunodeficiency syndrome and neurodegenerative diseases in humans and animals. Epidemiological studies [1]–[3] have indicated that the risk of dilated cardiomyopathy and non-suppurative myocarditis rises in those infected with human immunodeficiency virus type 1 (HIV-1). As experimental evidence in non-human primates, non-suppurative myocarditis and dilated cardiomyopathy with myocardial hypertrophy have been described in rhesus monkeys infected with simian immunodeficiency viruses (SIVs) [4]. These may occur as a result of the retrovirus itself acting either directly or indirectly via immunological mechanisms, opportunistic infection of other cardiotropic viruses or a combination of these mechanisms [1]–[4]. HIV viral protein R (Vpr) has been recently suggested to be a unique polypeptide that causes atrial cardiomyocyte mitosis, mesenchymal tumor and dysrhythmia in the heart of transgenic mice with Vpr [5]. However, the information on the real cause and pathogenesis of retrovirus-induced cardiac disorders is limited.

In animals other than humans and non-human primates, Maedi-Visna virus from the genus *Lentivirus* and subfamily *Orthoretrovirinae* induces primarily lymphocytic inflammation in the heart of sheep, but no alteration of cardiomyocytes [6]. In avian species, myocarditis associated with avian leukosis virus (ALV) belonging to *Alpharetrovirus* in the family *Retroviridae* has been reported in chickens [7]–[9]. ALVs in chickens are classified into six subgroups of A–E and J depending on envelope antigenicity, and subgroup A of ALV (ALV-A)-induced lymphoid leukosis is the most common neoplasm in infected birds. However, Rous-associated virus 1 (RAV-1) [8] and fowl glioma-inducing virus (FGV prototype) [9], both of which are ALV-A, could induce non-suppurative myocarditis in chickens and the former resulted in chronic circulatory disturbance. The myocarditis induced by ALV-A has been considered to be associated with excessive viral replication in cardiomyocytes [8], [9]. Additionally, dilated cardiomyopathy characterized by biventricular dilation and right ventricular hypertrophy has been described in chickens congenitally infected with ALV-J [10]. The authors speculated that the cardiomyopathy resulting from persistent high-level synthesis of viral products may usurp the cellular machinery and substrates in cardiomyocytes and Purkinje fibers. These reports imply that several strains of ALV are pathogenic to terminally differentiated cardiomyocytes. The causal relationship, however, is not completely established and the pathogenesis remains unclear. Here, we describe unusual hypertrophy and mitosis of cardiomyocytes, the morphology of which has not been previously reported in chickens infected with ALV-A. In addition, the causal relationship and pathogenesis are discussed on the basis of the molecular characteristics of isolates and the results of a reproducibility experiment with isolates.

## Materials and Methods

### Ethics statement

The Animal Care and Use Committee of Hokkaido University approved the protocols (Permit number 110071 and 120020), in accordance with the Act on Welfare and Management of Animals of Japanese government.

### Animals, histopathology and immunohistochemistry

The chicken breeds used in this study are listed in Table 1. These fowls were collected from the chicken population in which ALVs are widespread. Feather pulps and cloaca swabs were collected from 18 Japanese native chickens and stored at −80°C until use. For histological examination, the chickens were euthanized humanely according to a procedure approved by Hokkaido University. The heart and other organs, including the liver, spleen, kidneys, lungs and brain, were fixed in 20% neutral-buffered formalin, routinely processed and embedded in paraffin wax. Sections (4 µm) were cut and stained with hematoxylin and eosin. Masson's trichrome staining was performed in order to score myocardial fibrosis. Immunostaining using the labeled streptavidin-biotin method (Nichirei Corp., Tokyo, Japan) was performed. The primary antibodies used were polyclonal antibody against ALV common antigens (1∶5000, courtesy of Dr. K. Tsukamoto, Azabu University, Japan) and monoclonal antibodies against ARV p17 antigen (1∶50, Genesis Biotech Inc., Taipei, Taiwan), PCNA (no dilution, DAKO, Copenhagen, Denmark), phosphorylated Akt (p-Akt) (1∶100, Cell Signaling Technology, Danvers, MA) and phosphorylated tuberin (p-tuberin) (1∶100, Cell Signaling Technology). The polyclonal antibody against ALVs was generated by immunization of rabbits with Rous-associated virus-2, as previously described [11]. This antibody recognizes mainly gs proteins (p27) and glycoprotein 85 (gp85) of ALVs.

**Table 1:** Clinical signs, gross findings, cardiac histopathology and results of ALV isolation in chickens.

					Degree of cardiac lesions				Viral isolation f		
No.	Breed	Sex (M/F) a	Age (year)	Gross findings	Hypertrophied myocardium with atypical nucleus b	Non-suppurative myocarditis c	Fibrosis d	Matrix inclusion body e	Cloaca swab	Brain	Isolated strain
1	Japanese Bantam	M	2	Anemia	−	+	+	109	+	ND	Km_5880
2	Japanese Bantam	M	1	Atrophic comb	+++	+++	+	39	+	ND	Km_5960
3	Japanese Bantam	M	2	None	−	++	+	7	+	ND	Km_5622
4	Japanese Bantam	M	3	None	−	++	++	99	+	ND	Km_5967
5	Japanese Bantam	M	1	None	−	+++	+	5	+	ND	Km_6042
6	Japanese Bantam	F	1	Emaciation	−	+	+	6	+	ND	Km_5623
7	Japanese Bantam	F	3	Emaciation	−	+	+	1	+	ND	Km_5625
8	Japanese Bantam	F	4	Yolk peritonitis	+	++	++	57	+	ND	Km_5968
9	Japanese Bantam	F	1	Renal cyst	+	++	++	1	+	ND	Km_6045
10	Japanese Bantam	F	1	None	−	+	+	129	+	ND	Km_5621
11	Japanese Bantam	F	3	None	−	++	+	3	+	ND	Km_5624
12	Japanese Bantam	F	1	None	+	++	++	2	+	ND	Km_5843
13	Japanese Bantam	F	2	None	+	++	+	43	+	ND	Km_5852
14	Japanese Bantam	F	3	None	+++	+++	+++	27	+	ND	Km_5892
15	Japanese Bantam	F	1	None	−	+	+	49	ND	+	Km_5897
16	Japanese Bantam	F	3	None	−	+++	+	1	+	ND	Km_6209
17	Sebright Bantam	F	3	None	++	++	++	19	ND	+	Km_5900
18	Kumamoto Long Tail	F	4	None	−	+++	++	12	+	ND	Km_6039

a M: male, F: female

b The degree of hypertrophied cardiomyocytes with atypical nuclei was defined as follows: +, individual cells; ++, multiple and/or small clusters; +++, multiple and extensive growth.

c The degree of non-suppurative myocarditis was defined as follows: +, focal or multifocal (under 25 µm in diameter); ++, multifocal (from 25 µm to 200 µm); +++, multifocal (over 200 µm).

d The degree of fibrosis was defined as follows: +, interstitial; ++, multifocal interstitial (under 400 µm in diameter); +++, multifocal interstitial (over 400 µm in diameter).

e Total number in 10 high-power fields.

f +: positive,−: negative,ND: no data.

### RNA extraction and cDNA synthesis

Total RNA was extracted from brains, kidneys and hearts using TRIzol reagent (Invitrogen Life Technologies, Carlsbad, California, USA). Reverse transcription (RT) was performed as previously described [12]. As an internal control for RNA extraction and cDNA synthesis, PCR amplification was performed for all cDNA samples using primers specific to chicken β-actin. The PCR for chicken β-actin was performed according to conditions described previously [12].

### PCR amplification

Two types of PCR amplification were carried out with cDNA using primer sets shown in Table 2. To detect the ALV genome, ALV-specific PCR was performed with a primer set of ALV #38 and ALV #39 according to conditions described previously [13]. The primers were designed for *env*SU (gp85), which is well conserved in ALV subgroups A to E. To detect the avian reovirus (ARV) genome, PCR for the S2 and S4 regions of ARV was performed with the primer sets of ARV sense primer (S2) and ARV antisense primer (S2), and ARV sense primer (S4) and ARV antisense primer (S4), respectively, using a procedure previously described with some modification [14]. The PCR mixture was a 10 µl reaction volume containing 1 µl of 10x *Ex Taq* Buffer (TaKaRa Bio, Hotsu, Japan), 0.8 µl of dNTP mixture, 2.5 mM each dNTP (TaKaRa Bio), 5 pmol each primer, 0.25 U *TaKaRa Ex Taq* HS (TaKaRa Bio) and 10 ng DNA. Touchdown PCR was performed. Denaturation was at 94°C for 1 min. The annealing temperature decreased in steps of 2°C, from 60°C down to 48°C. Two cycles were performed at each annealing temperature. Thirty-five cycles were then carried out at 95°C for 30 sec, 48°C for 30 sec and 72°C for 30 sec. Finally, a prolonged extension step was performed at 72°C for 10 min. The PCR products were analyzed by 2% agarose gel electrophoresis.

**Table 2:** Sequence of the oligonucleotide primers and targets.

Primer	Sequence (5′-3′)	Accession No.
Chicken β-actin forward	TAT CCG TTA GGA TCT GTA TG	L08165
Chicken β-actin reverse	ATC TCG TCT TGT TTT ATG CG	L08165
ALV #38	TTA GGT TCC CAG TCT CTC CC	AB112960
ALV #39	ATT GCG GGT GGT AGC GCT TT	AB112960
ARV sense primer (S2)	CCC ATG GCA ACG ATT TC	AF104311
ARV antisense primer (S2)	TTC GGC CAC GTC TCA AC	AF104311
ARV sense primer (S4)	GTG CGT GTT GGA GTT TC	U95952
ARV antisense primer (S4)	ACA AAG CCA GCC ATG AT	U95952

### Virus isolation and identification

For virus isolation, the supernatants of cloaca swabs from 16 chickens were inoculated with 80% confluent DF-1 cells. DF-1 cells were obtained from the American Type Culture Collection (Manassas, Virginia, USA) and cultured as previously described. Cells were harvested 7 days after inoculation and were collected at that time. Concentrated culture supernatant was prepared according to the methods described previously [15].

### Sequence analysis and phylogenic analysis

The *env*SU regions of 15 strains isolated from field cases and the complete virus genome of Km_5892 were sequenced as previously described [12], [16]. The complete genomic RNA (gRNA) of Km_5892 has been deposited in the DDBJ/GenBank (AB682778). The obtained sequences were analyzed with the Clustal W 1.8.3 program on the DDBJ website (http://clustalw.ddbj.nig.ac.jp/top-j.html) and the NCBI BLAST program (http://blast.ncbi.nlm.nih.gov/Blast.cgi). Phylogenic trees based on nucleotide sequences of *env*SU and *pol* were constructed on the DDBJ website by the neighbor-joining method implementing the Kimura method using isolated virus and other ASLVs. The topological accuracy of the tree was estimated by the bootstrap method with 1000 replicates.

### Birds and experimental infection

Fertile eggs from specific-pathogen-free (SPF) White Leghorn strain WL-M/O (C/O) chickens were used for experimental infection. The fertile eggs were obtained from Nippon Institute for Biological Science (Yamanashi, Japan). This strain lacks both chicken helper factor and group-specific antigen and is susceptible to ALV subgroups A to E (International Registry of Poultry Genetic Stocks, Bulletin 476, March 1988, Ralph G. Somes, Jr., University of Connecticut, Storrs, Connecticut, USA). The presence of endogenous virus genes other than chicken helper factor and group-specific antigen is not known. Chicken embryos were inoculated via the yolk sac on the sixth day of incubation with 5×10^4^ Tissue Culture Infectious Units (TCIUs) of Km_5666 or Km_5892 strain and were euthanized at 35, 70 and 140 days of age. Seven control chicken embryos belonging to the uninfected group were similarly inoculated with 0.1 ml uninfected tissue culture medium and were euthanized at 35 days and 70 days of age. The hatched chickens were reared humanely according to the guidelines set by the Animal Care and Use Committee of our institute. No chickens received any vaccinations or medication.

## Results

### Clinical signs and pathology in Japanese native fowls

**Figure 1:**
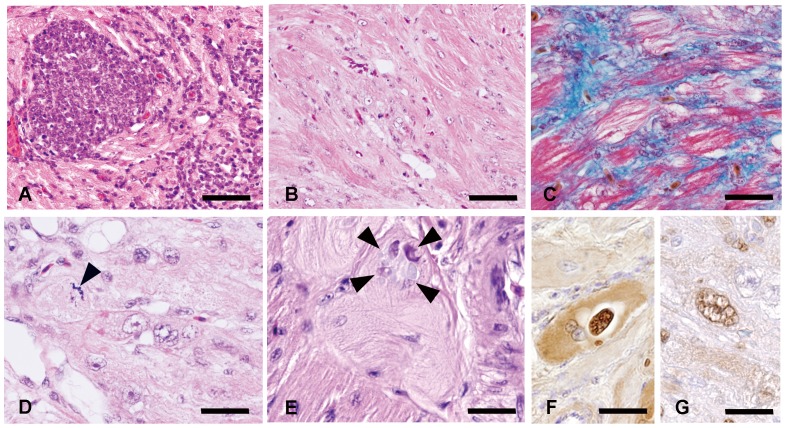
Histopathology and immunohistochemistry of hearts in Japanese native fowls. (A) Mononuclear cells infiltrate into myocardial fibers. Chicken No. 5. Bar  = 40 µm. (B) Disarrangement of myocardial fibers with interstitial fibrosis. Chicken No. 9. Bar  = 90 µm. (C) Interstitial myocardial fibrosis confirmed by Masson's trichrome stain. Chicken No. 14. Bar  = 25 µm. (D) Hypertrophied cardiomyocytes with multiple atypical nuclei. Mitosis (arrowhead) is occasionally noted in these cardiomyocytes. Chicken No. 14. Bar  = 20 µm. (E) Matrix inclusion bodies (arrowhead) in the cytoplasm of Purkinje fibers. Chicken No. 14. Bar  = 20 µm. (F) Purkinje fibers and matrix inclusion bodies positive for ALV common antigen. Chicken No. 14. Bar  = 20 µm. (G) A large atypical nucleus in a hypertrophied cardiomyocyte is positive for PCNA. Chicken No. 14. Bar  = 20 µm.

Eighteen Japanese native fowls were collected and examined. A low rate of egg laying was observed in four fowls and emaciation in two (Table 1). At necropsy, one bird each had a small renal cyst and egg yolk in the body cavity (yolk peritonitis). Other twelve fowls showed no apparent gross lesions. The main histopathological changes in hearts were lymphocytic myocarditis, matrix inclusion bodies in cardiomyocytes and Purkinje fibers, fibrosis and hypertrophy of cardiomyocytes with atypical nuclei (Figure 1A–D). These lesions were noted mainly in the left ventricular wall and the degree of severity varied (Table 1). Multifocal lymphocytic myocarditis with mild myocardial necrosis and mild to severe interstitial myocardial fibrosis were observed in all cases. These foci contained a small number of plasma cells and macrophages. Intracytoplasmic, round to oval, unstained to basophilic inclusions (matrix inclusion bodies) were detected in cardiomyocytes and Purkinje fibers of all cases (Figure 1E). Additionally, individual hypertrophied cardiomyocytes or small clusters of cardiomyocytes with atypical, large nuclei were observed in seven (39%) fowls and these cardiomyocytes occasionally contained matrix inclusion bodies. The atypical nuclei were frequently arranged in long chains (1–11/high-power field) in the cytoplasm, and mitotic figures were also observed in these cardiomyocytes. The degree of cardiomyocyte hypertrophy was not correlated to the degree of myocarditis and the frequency of matrix inclusion bodies in the cytoplasm (Table 1). Other eleven chickens had neither hypertrophied cardiomyocytes nor atypical nucleus. Ten (56%) of eighteen fowls developed gliomas in their brains. No significant lesion was observed in other organs of the examined cases, except for a renal cyst and yolk peritonitis grossly noted in one bird each. These cardiomyocytes, Purkinje fibers and matrix inclusion bodies were immunohistochemically positive for ALV common antigen (Figure 1F) and negative for avian reovirus (ARV) p17 antigen. Nuclei in hypertrophied cardiomyocytes were positive for proliferating cell nuclear antigen (PCNA) (Figure 1G).

### Viral isolation and polymerase chain reaction for ALV and ARV

Feather pulps from all cases showed positivity for ALV-specific polymerase chain reaction (PCR) and ALVs were isolated from cloaca swabs or brains of all affected chickens (Table 1). The hearts from 5 chickens (No. 2, 5, 9, 14 and 16), 2 chickens (No. 2 and 14) and the kidneys from other 13 birds showed negativity for ARV-specific PCR.

### Phylogenic analysis of isolated ALV strains

**Figure 2:**
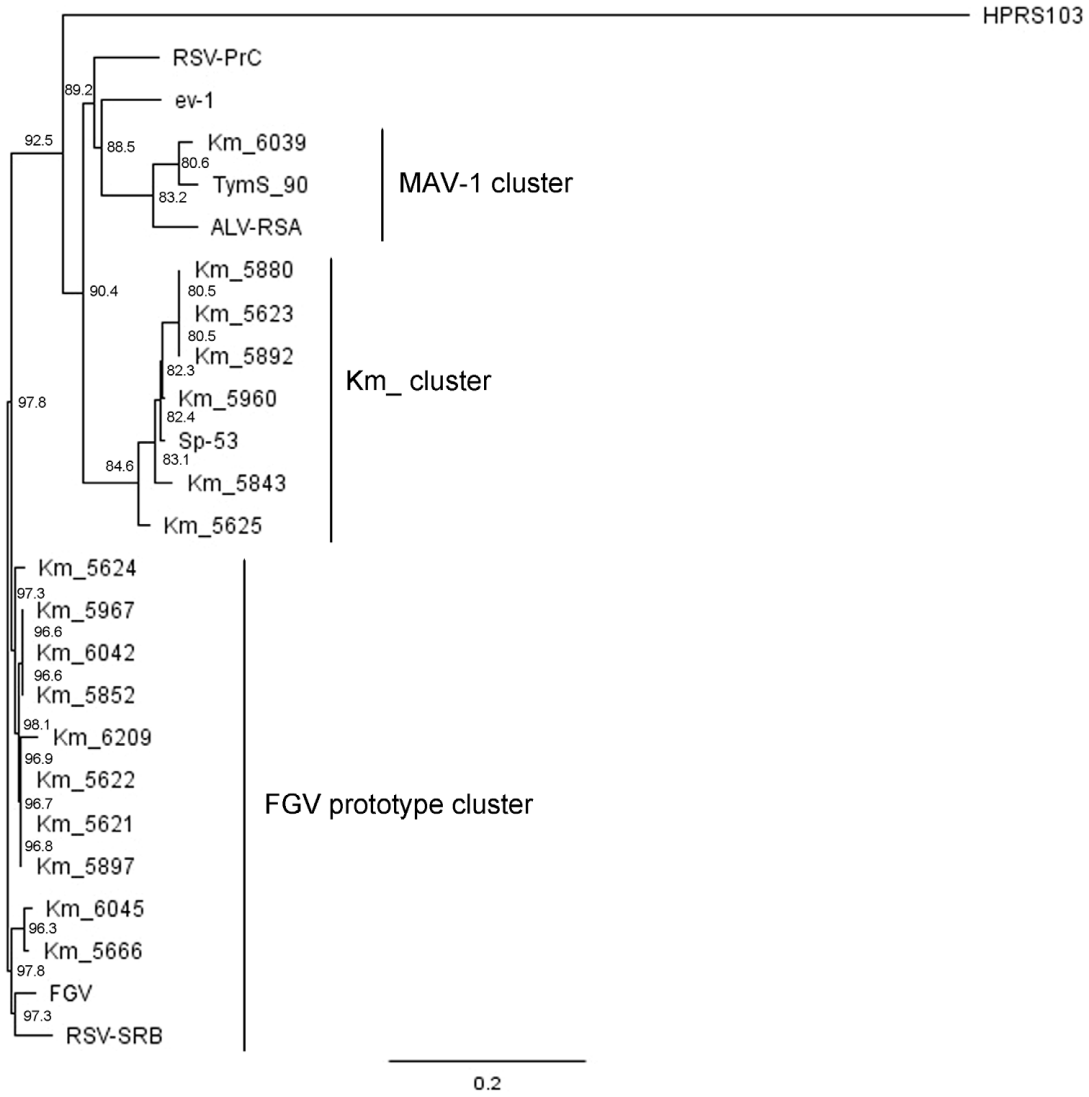
Phylogenic tree based on *env*SU region of the isolates. Phylogenic tree constructed by the neighbor-joining method showing the relationships among the isolates, FGV, Sp-53 (FGV variant) and standard ALSVs based on the SU region of the *env* gene. The amino acid sequences were aligned with the Clustal W 1.8.3 program. Bootstrap values of 1000 trials using the neighbor-joining method. Scale bar corresponds to a distance of 0.2 as the frequency of amino acid substitutions in the pairwise comparison of two sequences according to the Kimura two-parameter method.

**Figure 3:**
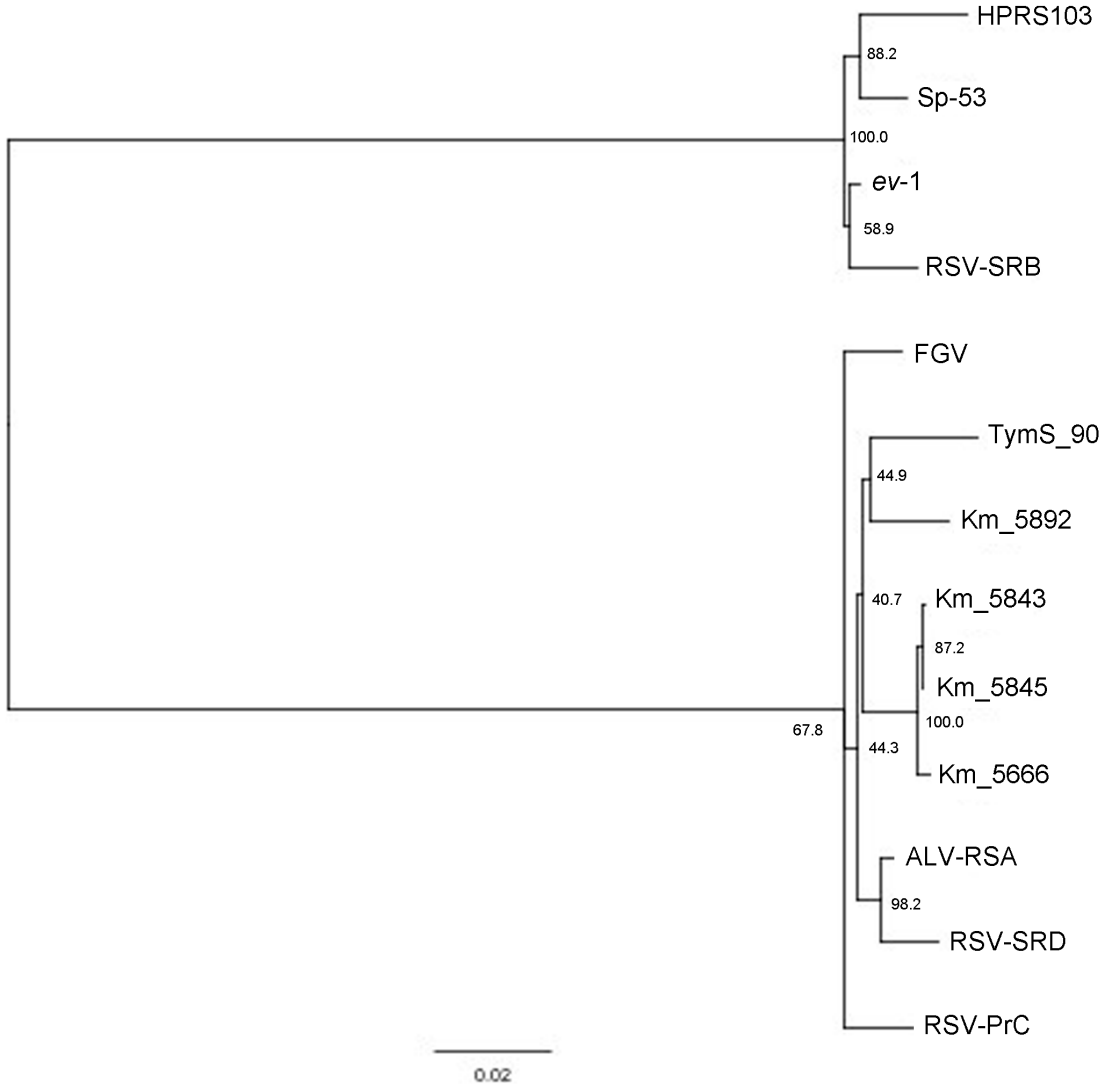
Phylogenic tree based on *pol* gene of the isolates. Phylogenic tree constructed by the neighbor-joining method showing the relationships among Km_5892, FGV prototype and standard ALSVs based on the *pol* gene. The nucleotide sequences were aligned with the Clustal W 1.8.3 program. Bootstrap values of 1000 trials using the neighbor-joining method. Scale bar corresponds to a distance of 0.03 as the frequency of amino acid substitutions in the pairwise comparison of two sequences according to the Kimura two-parameter method.

The sequences of *env*SU genes of sixteen strains isolated from the affected chickens were determined. The phylogenic analysis based on this region revealed that these strains could be classified into three clusters: MAV-1, FGV prototype and Km_ clusters (Figure 2). Five strains, including Km_5843, Km_5852, Km_5892, Km_5960 and Km_6045, isolated from fowls with hypertrophied cardiomyocytes belonged to two different clusters: FGV prototype and Km_ clusters. Next, the full-length genome of Km_5892, an isolate from the fowl with the most extensive distribution of hypertrophied cardiomyocytes, was determined. The *env* gene of Km_5982 showed homology of 97% with that of FGV mutant strain Sp-40, and the 5′UTR, *gag-pol* and 3′LTR domains of Km_5982 showed 96%, 96% and 90% homology with those of TymS_90, respectively. Phylogenic analysis based on the *pol* gene revealed that Km_5892 could be classified into the same cluster as the other Km_ strains, including Km_5666, Km_5843 and Km_5845 (Figure 3). Km_5666 (accession number AB669896) is an ALV-A that was isolated from a Japanese Bantam kept in the same flock and the sequence of its full-length genome was previously analyzed [17].

### Pathology of chickens experimentally inoculated with Km_strains

Km_5666 and Km_5892 were picked up from two different clusters in phylogenic tree based on *env*SU region of ALV and we performed infectious experiment using these two strains. C/O SPF chicken eggs, inoculated *in ovo* with 5×10^4^ TCIU of Km_5666 and Km_5892 on the sixth day of incubation, were euthanized at 35, 70 and 140 days of age and examined pathologically (Table 3).

**Table 3:** The degree of cardiac lesions in chickens experimentally infected with Km_5666 and Km_5892.

			Hypertrophied myocardium with atypical nucleus^a^				Non-suppurative myocarditis^b^				Fibrosis^c^		Matrix inclusion body^d^	
Inoculated strain	Age (days)	N	−	+	++	+++	−	+	++	+++	−	+	−	+
Km_5666	35	6	5	0	0	1	2	3	0	1	6	0	5	1
	70	3	2	1	0	0	0	1	2	0	3	0	1	2
	140	3	3	0	0	0	0	1	2	0	2	1	1	2
Km_5892	35	4	4	0	0	0	0	2	2	0	3	1	0	4
	70	3	2	0	1	0	0	1	2	0	2	1	0	3

a The degree of hypertrophied cardiomyocytes with atypical nuclei was defined as follows: +, individual cells; ++, multiple and/or small clusters; +++, multiple and extensive growth.

b The degree of non-suppurative myocarditis was defined as follows: +, focal or multifocal (under 25 µm in diameter); ++, multifocal (from 25 µm to 200 µm); +++, multifocal (over 200 µm).

c The degree of fibrosis was defined as follows: +, interstitial; ++, multifocal interstitial (under 400 µm in diameter); +++, multifocal interstitial (over 400 µm in diameter).

d Total number in 10 high-power field.

**Figure 4:**
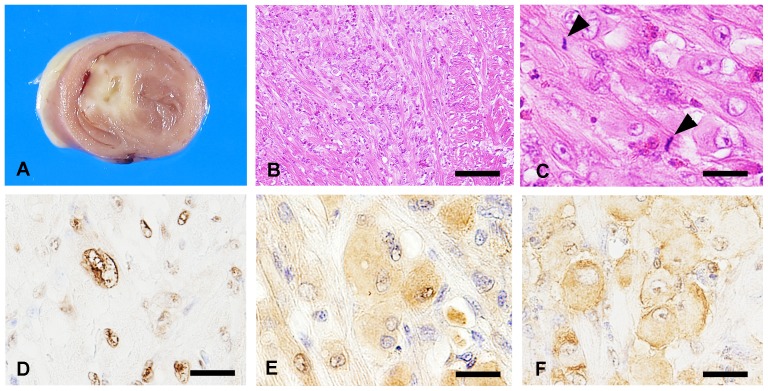
Gross pathology, histopathology and immunohistochemistry of chickens experimentally inoculated with Km_5666. (A) Grayish-white ill-defined tissue was located in the septum at 35 days old. (B) Locally extensive growth of hypertrophied cardiomyocytes. Bar  = 75 µm. (C) Mitosis (arrowhead) observed in hypertrophied cardiomyocytes. Bar  = 30 µm. (D) Numerous nuclei in atypical myocardium were positive for PCNA. Bar  = 20 µm. (E–F) Hypertrophied cardiomyocytes positive for p-Akt (E) and p-tuberin (F). Bars  = 20 µm.

Paralysis of the left leg was observed in one of three (33%) Km_5666-inoculated chickens at 70 days of age. Other chickens showed no clinical signs. Multifocal lymphocytic myocarditis was observed in ten (83%) of twelve birds inoculated with Km_5666 and seven (100%) of seven with Km_5892. In these foci, lymphocytes and plasma cells admixed with a few macrophages and heterophils also infiltrated to various degrees. Hypertrophied cardiomyocytes with atypical nuclei were observed in two (17%) chickens with Km_5666 and one (14%) with Km_5892. One of the Km_5666-inoculated birds showed extensive multiple growths of atypical cardiomyocytes at 35 days of age (Figure 4A–C). Interstitial myocardial fibrosis associated with the inflammatory foci was observed in one (8%) Km_5666-inoculated chicken and two (29%) Km_5892-inoculated birds. Matrix inclusion body was detected in five (42%) and seven (100%) chickens inoculated with Km_5666 and Km_5982, respectively. In terms of immunohistochemistry, the cytoplasm of cardiomyocytes and Purkinje fibers and matrix inclusion bodies were positive for ALV common antigen and nuclei in atypical cardiomyocytes were positive for PCNA (Figure 4D). The cytoplasm of hypertrophied cardiomyocytes was also positive for phosphorylated Akt (p-Akt) and phosphorylated tuberin (p-tuberin) (Figure 4E–F). By using RT-PCR and sequencing, the hypervariable regions of Km_5666 and Km_5982 were detected from the brain and heart of inoculated birds, respectively. No significant lesion was observed in the control birds inoculated with medium.

## Discussion

The cardiac lesions of native fowls were histologically characterized by atypical hypertrophied cardiomyocytes with mitosis and non-suppurative myocarditis. ALV strains were isolated from all affected cases and similar cytological changes were reproduced by experimental infection with Km_5666 and Km_5982. In addition, extensive multiple growths of atypical cardiomyocytes were also recognized in a Km_5666-inoculated bird. These results demonstrate that ALVs can cause atypical hypertrophied cardiomyocytes and mitosis and on rare occasions induce extensive growth. The distribution and cytological features of cardiomyocytes clearly differed from those of physiological cardiac hypertrophy and those of idiopathic primary myocardial diseases, including human and feline cardiomyopathies [18]–[20]. To our knowledge, such individual and/or massive hypertrophy of cardiomyocytes has not been previously reported in any avian disease, except for in one chicken experimentally infected with FGV [21].

ARV causes a cardiac abnormality grossly characterized by right atrial dilation and microscopically non-suppurative myocarditis with multifocal necrosis and multinuclear giant cell formation [22]. However, ARV infection occurs mainly in turkeys rather than in chickens, and the histological changes were easily differentiated from those of our cases,especially on the basis of atypical hypertrophy of cardiomyocytes. In addition, our cases were immunohistochemically negative for ARV p17 antigen and negative for ARV S2 and S4 genome by PCR.

Cardiac rhabdomyomas are often found incidentally in human infants affected by tuberous sclerosis [23] and in swine juveniles [24], and are considered as a malformation or a hamartoma rather than a true neoplasm [18]–[20]. Rhabdomyomas are morphologically characterized by multiple nodules composed of enlarged cardiomyocytes with a distinct border and the appearance of “spider cells”, which are defined as large cardiomyocytes with numerous vacuoles in their cytoplasm due to glycogen accumulation. In contrast, their malignant counterpart, rhabdomyosarcoma, is quite rare in avian species and there has been just one report of rhabdomyosarcoma induced by the MC29 strain [25], [26], which is a replication-deficient avian myelocytomatosis virus with v-*myc* oncogene and belongs to the group of acutely transforming viruses. Morphologically, rhabdomyosarcoma is characterized by the proliferation of large vacuolated cardiomyocytes with severe cellular and nuclear atypia and shows invasive growth. Hypertrophied cardiomyocytes showing extensive growth in a Km_5666-inoculated bird had glassy cytoplasm with a few vacuoles. Although these morphological features were different from those of naturally occurring cardiac neoplasms, the cardiac lesions consisting of atypical cardiomyocytes in a Km_5666-inoculated chick (35 days of age) were considered to be in the category of hamartoma.

ALVs show high tropism to myocardial fibers and matrix inclusion bodies are frequently formed in association with ALV replication in infected cardiomyocytes [27]–[29]. Non-suppurative myocarditis was found to occur in experimental cases with some ALV strains, including RAV-1 and 7 [7], [30], and these inflammatory changes are interpreted as being induced by viral replication in cardiomyocytes. On the basis of these findings, four possible mechanisms are considered to explain the ALV-induced cardiomyocyte hypertrophy in the present study. First, these lesions may develop as reactive changes secondary to non-suppurative myocarditis. The frequency of hypertrophied cardiomyocytes, however, was not correlated with the degree of myocarditis and we have never experienced such changes in any myocarditis cases in chickens. It seems unlikely that the abnormal cardiomyocytes are merely a reactive change. Second, the lesions may develop due to physical stimuli of matrix inclusion bodies. Hypertrophy of myocardial fibers is known to be induced by the formation of matrix inclusion bodies [10]. However, there are no findings that atypical large nuclei and mitosis develop in association with the formation of matrix inclusion bodies in any previous reports on ALV-infected heart. Third, the formation of atypical cardiomyocytes may be associated with insertional mutagenesis of ALV [31]. However, transformation of host cells usually take more than 5 months in avian lymphoid leukosis by ALV-A because multiple rounds of infection must occur in an infected animal before a provirus inserts itself in the vicinity of a cellular proto-oncogene with appropriate activation. Insertional mutagenesis cannot explain the development of cardiomyocyte hypertrophy and hamartoma in 35-day-old birds in this study. Three oncogenic mechanisms of retroviruses other than insertional mutagenesis are known: 1) transformation by viral oncogenes, 2) oncogenesis by accessory proteins such as Tax of human T-cell leukemia virus type I and 3) envelope protein-induced transformation [31]. The viral genomes of Km_5666 [11] and Km_5892, however, contain neither a viral oncogene nor any genes coding accessory proteins. The last possible mechanism is that viral components such as envelope proteins may cause signaling pathway abnormality in host cells, resulting in a change of cellular function and morphology [31]. Recently, Jaagsiekte sheep retrovirus (JSRV) has been reported to cause transformation of host cells by its transmembrane (TM) envelope domain [32], [33]. Envelope protein of JSRV involves the PI3K/Akt pathway in transformation [33]. Once the PI3K/Akt pathway is activated, cellular genes associated with protein synthesis, proliferation and survival are up-regulated. Among these genes, tuberin and mammalian target of rapamycin (mTOR) are known to have a crucial role in the proliferation and hypertrophy of cells [34]. Dephosphorylated tuberin (activated form) was shown to regulate cellular status by suppressing the mTOR activator, Rheb. In the presence of stimulation, phosphorylated Akt (p-Akt) suppresses the activation of tuberin by phosphorylation, resulting in cellular hypertrophy and proliferation. Immunohistochemical results on p-tuberin and p-Akt in this study suggest that hypertrophy and proliferation of cardiomyocytes may be caused by activation of the mTOR signaling pathway.

The *env* gene and LTR are mainly involved in the tumorigenesis of ALVs. A previous study indicated that the *env* gene of Km_5666 is very similar to the FGV prototype, whereas other regions of this strain, including 5′UTR, *gag-pol*, 3′UTR and 3′LTR, show high homology with TymS_90 [17]. On the other hand, the *env* gene of Km_5892 was found to be closely related to that of FGV variant (Sp-40) [35], whereas other regions, including 5′UTR, *gag-pol* and 3′LTR, were shown to be similar to those of TymS_90. Km_5666 and the other isolates from birds affected by cardiomyocyte hypertrophy in the current study belonged to two clusters in the phylogenic analysis based on the *env*SU region. These results suggest that there is genome diversity among ALVs showing the inducibility of cardiomyocyte hypertrophy, implying a high prevalence of cardiac abnormality among Japanese native fowls infected with ALV.

Several reports have indicated the possibility that retroviruses have a direct role in the pathogenesis of myocarditis and dilated cardiomyopathy in humans, monkeys and other animals [3]–[9], although the relationship between retrovirus infection and cardiac abnormality remains obscure. Whether HIV-1 and SIV can infect cardiomyocytes is still controversial [36], [37], whereas the fact that ALV has strong tropism for cardiomyocytes and Purkinje fibers is evidenced by the frequent formation of matrix inclusion bodies in these cells [27]–[29]. Despite these different degrees of tropism for cardiomyocytes, ALV-induced myocardial alteration is considered to be useful for clarifying the pathogenesis of retroviral cardiomyopathy.

In conclusion, our results indicate that several strains of ALV could promote growth activity of cardiomyocytes and induce cardiomyocyte hypertrophy by a mechanism other than insertional mutagenesis. This is the first report showing unique hypertrophy and growth of cardiomyocytes induced by ALV. This animal model may provide new insight into retrovirus-induced cardiac pathology.
